# Chemistry and Biology of Noncanonical Nucleic Acid Structures: From Physicochemical Properties to Therapeutic Applications

**DOI:** 10.3390/ijms25094952

**Published:** 2024-05-01

**Authors:** Jussara Amato, Antonio Randazzo, Bruno Pagano

**Affiliations:** Department of Pharmacy, University of Naples Federico II, 80131 Napoli, Italy; jussara.amato@unina.it (J.A.); antonio.randazzo@unina.it (A.R.)

The aim of this Special Issue is to highlight significant and new aspects concerning the chemistry and biology of noncanonical nucleic acid structures, with emphasis on their structure, stability, and conformational equilibria, as well as on the biological relevance of their interactions with proteins and ligands.

Nucleic acids have the ability to form several different hydrogen bonding patterns, enabling them to attain various structural and conformational polymorphic forms [1]. These include single-stranded forms and secondary structures like the canonical double helix, as well as noncanonical structures such as triplex, G-quadruplex, and i-motif species [2]. Noncanonical structures may, in turn, encompass an ensemble of conformers depending on the sequence composition and/or environmental conditions [3,4]. In vivo, noncanonical nucleic acid structures can form in important genomic regions and play key roles in several biological processes, including the modulation of oncogenes expression and telomere maintenance [5,6,7,8]. Consequently, they represent potential anticancer drug targets [9,10,11].

Many nucleic acid aptamers, characterized by their propensity to adopt specific structural conformations, also fold into noncanonical structures, thus forming unique three-dimensional architectures capable of specifically recognizing their targets at the molecular level [12,13]. Moreover, the nanoscale geometry and dynamic properties of noncanonical nucleic acid structures, along with their inherent biocompatibility, make them promising candidates for the development of functional nanodevices [14,15].

The investigations published in this Special Issue cover many of these aspects. Indeed, one of them provides insight into the promoter region of the oncogene *MST1R* [16], which encodes for a receptor belonging to the epithelial–mesenchymal transition factor family involved in the formation of metastases. The *MST1R* promoter is enriched in guanine residues that can potentially form G-quadruplexes to regulate gene transcription, as observed in other oncogene promoters such as *KRAS* and *c-MYC* [7,17,18]. In this work, the reverse and forward sequences of the *MST1R* promoter region were studied using a bioinformatics tool and biophysical methods were used to characterize the best scored sequences, thus highlighting their potential role in transcription regulation.

Another contribution investigates the selective targeting of cancer-related G-quadruplex structures by natural compounds and identifies the alkaloid dicentrine as a promising G-quadruplex ligand with anticancer activity [19].

Libera et al. utilized UV resonance Raman scattering to explore the vibrational behavior of a human telomeric G-quadruplex and its aqueous solvent during the thermal denaturation of the DNA molecule [20]. The results revealed that in aqueous G-quadruplex solutions, as in canonical B-DNA, the solute–solvent interactions detected by Raman spectroscopy are mutual. Intriguingly, water molecular vibrations were further associated with thermally induced topological changes at the secondary structure level. Due to the importance of G-quadruplexes for a variety of biological functions, the results achieved could be a starting point for further investigations aimed at improving the current knowledge on the impact of the molecular environment on the conformation of the DNA.

Additionally, one manuscript provides a detailed biophysical characterization and in vitro and in vivo biological studies of a G-quadruplex-forming aptamer that specifically binds to the mutant huntingtin protein [21], a privileged target of many cutting-edge pharmacological strategies to combat Huntington’s disease, a serious neurodegenerative disease for which only limited palliative treatments are currently available.

Aviñó et al. propose an innovative analytical method named TENADA (triplex enhanced nucleic acid detection assay) for the detection of SARS-CoV-2 RNA [22]. This strategy uses polypurine reverse-Hoogsteen hairpin oligonucleotides that form high-affinity triplexes with the SARS-CoV-2 polypyrimidine sequences for an efficient capture of the viral genome, and a second labeled oligonucleotide is used to detect the formation of a trimolecular complex similar to antigen tests. In this way, the presence of SARS-CoV-2 RNA is detected by the formation of a ternary complex on a biosensor surface. In principle, the TENADA assay can be easily adapted for the detection of any pathogen, requiring only knowledge of its genome sequence, demonstrating the potential of noncanonical DNA structures for improving biomedical applications.

The studies published in this Special Issue, along with others, demonstrate how fascinating and complex the world of noncanonical nucleic acid structures is. There is still much to learn about noncanonical DNA/RNA structures. In particular, the dynamic and transient nature of these diverse non-B-DNA structures in the cellular molecularly crowded environment has always been a challenge for targeting or recognizing these structures.

From a physicochemical perspective, the formation mechanism of nucleic acid structures is mainly governed by interactions with cations and water molecules, leading to the formation of H-bonds and stacking interactions. Considering the interplay with the surrounding environment, nucleic acids bind to cations to mitigate electrostatic repulsion caused by negatively charged phosphate groups. Specifically, noncanonical structures are stabilized by the binding of certain ions [23]. For instance, increasing the concentrations of H^+^ and Mg^2+^ ions in a solution stabilizes the triplexes [24], whereas elevated levels of K^+^ ions specifically stabilize G-quadruplexes. Notably, pathological conditions like cancer can lead to changes in ion concentrations resulting from the overexpression or inactivation of ion channel proteins specific to the disease [25]. Additionally, the overexpression of certain proteins may diminish the water activity in the solution, thereby further stabilizing noncanonical structures [26]. Consequently, elucidating the mechanisms governing the formation of noncanonical nucleic acid structures may offer new perspectives and insights into the mechanisms underlying the onset of diseases. This, in turn, could lead to the development of strategies to modulate the structures and stabilities of nucleic acids in disease-associated genes for therapeutic purposes.

Noncanonical nucleic acids also offer significant opportunities as therapeutic agents to expand the plethora of classical therapeutic targets, from extracellular and surface proteins to intracellular regulators, in a wide range of diseases. Indeed, therapeutic nucleic acids like aptamers can potentially inhibit molecular interactions and modulate or induce specific biological processes [12,27]. A common thread of nucleic acid aptamers with noncanonical structures, which makes them very promising for therapeutic applications, is their uniqueness, flexibility, and binding specificity. For example, some aptamers bind to misfolded target proteins and impede their accumulation in the central nervous system, thus showing promise in treating neurodegenerative diseases including Alzheimer’s disease, Parkinson’s disease, Huntington’s disease, and prion diseases [28,29,30].

In conclusion, the structures and functions of noncanonical DNA and RNA continue to present many surprises. Discussions about them foster an ever-growing interest in the paradigm of noncanonical structural polymorphism of nucleic acids, stabilization, and their implication in understanding the role in different biological processes.

## References

[1] Rich A. (1993). DNA Comes in Many Forms. Gene.

[2] Sugimoto N., Endoh T., Takahashi S., Tateishi-Karimata H. (2021). Chemical Biology of Double Helical and Non-Double Helical Nucleic Acids: “To B or Not To B, That Is the Question”. Bull. Chem. Soc. Jpn..

[3] Choi J., Majima T. (2011). Conformational Changes of Non-B DNA. Chem. Soc. Rev..

[4] Jana J., Weisz K. (2021). Thermodynamic Stability of G-Quadruplexes: Impact of Sequence and Environment. ChemBioChem.

[5] Makova K.D., Weissensteiner M.H. (2023). Noncanonical DNA Structures Are Drivers of Genome Evolution. Trends Genet..

[6] Duardo R.C., Guerra F., Pepe S., Capranico G. (2023). Non-B DNA Structures as a Booster of Genome Instability. Biochimie.

[7] Romano F., Di Porzio A., Iaccarino N., Riccardi G., Di Lorenzo R., Laneri S., Pagano B., Amato J., Randazzo A. (2023). G-Quadruplexes in Cancer-Related Gene Promoters: From Identification to Therapeutic Targeting. Expert Opin. Ther. Pat..

[8] Zizza P., Cingolani C., Artuso S., Salvati E., Rizzo A., D’Angelo C., Porru M., Pagano B., Amato J., Randazzo A. (2016). Intragenic G-Quadruplex Structure Formed in the Human CD133 and Its Biological and Translational Relevance. Nucleic Acids Res..

[9] Neidle S. (2017). Quadruplex Nucleic Acids as Targets for Anticancer Therapeutics. Nat. Rev. Chem..

[10] Amato J., Miglietta G., Morigi R., Iaccarino N., Locatelli A., Leoni A., Novellino E., Pagano B., Capranico G., Randazzo A. (2020). Monohydrazone Based G-Quadruplex Selective Ligands Induce DNA Damage and Genome Instability in Human Cancer Cells. J. Med. Chem..

[11] Marzano S., Miglietta G., Morigi R., Marinello J., Arleo A., Procacci M., Locatelli A., Leoni A., Pagano B., Randazzo A. (2022). Balancing Affinity, Selectivity, and Cytotoxicity of Hydrazone-Based G-Quadruplex Ligands for Activation of Interferon β Genes in Cancer Cells. J. Med. Chem..

[12] Zhou J., Rossi J. (2017). Aptamers as Targeted Therapeutics: Current Potential and Challenges. Nat. Rev. Drug Discov..

[13] Brown A., Brill J., Amini R., Nurmi C., Li Y. (2024). Development of Better Aptamers: Structured Library Approaches, Selection Methods, and Chemical Modifications. Angew. Chemie Int. Ed..

[14] Yatsunyk L.A., Mendoza O., Mergny J.-L. (2014). “Nano-Oddities”: Unusual Nucleic Acid Assemblies for DNA-Based Nanostructures and Nanodevices. Acc. Chem. Res..

[15] Mergny J.-L., Sen D. (2019). DNA Quadruple Helices in Nanotechnology. Chem. Rev..

[16] Robert C., Marquevielle J., Salgado G.F. (2022). The Promoter Region of the Proto-Oncogene MST1R Contains the Main Features of G-Quadruplexes Formation. Int. J. Mol. Sci..

[17] Cogoi S., Xodo L.E. (2006). G-Quadruplex Formation within the Promoter of the KRAS Proto-Oncogene and Its Effect on Transcription. Nucleic Acids Res..

[18] Siddiqui-Jain A., Grand C.L., Bearss D.J., Hurley L.H. (2002). Direct Evidence for a G-Quadruplex in a Promoter Region and Its Targeting with a Small Molecule to Repress c-MYC Transcription. Proc. Natl. Acad. Sci. USA.

[19] Platella C., Ghirga F., Musumeci D., Quaglio D., Zizza P., Iachettini S., D’Angelo C., Biroccio A., Botta B., Mori M. (2023). Selective Targeting of Cancer-Related G-Quadruplex Structures by the Natural Compound Dicentrine. Int. J. Mol. Sci..

[20] Libera V., Bianchi F., Rossi B., D’Amico F., Masciovecchio C., Petrillo C., Sacchetti F., Paciaroni A., Comez L. (2022). Solvent Vibrations as a Proxy of the Telomere G-Quadruplex Rearrangements across Thermal Unfolding. Int. J. Mol. Sci..

[21] Riccardi C., D’Aria F., Digilio F.A., Carillo M.R., Amato J., Fasano D., De Rosa L., Paladino S., Melone M.A.B., Montesarchio D. (2022). Fighting the Huntington’s Disease with a G-Quadruplex-Forming Aptamer Specifically Binding to Mutant Huntingtin Protein: Biophysical Characterization, In Vitro and In Vivo Studies. Int. J. Mol. Sci..

[22] Aviñó A., Cuestas-Ayllón C., Gutiérrez-Capitán M., Vilaplana L., Grazu V., Noé V., Balada E., Baldi A., Félix A.J., Aubets E. (2022). Detection of SARS-CoV-2 Virus by Triplex Enhanced Nucleic Acid Detection Assay (TENADA). Int. J. Mol. Sci..

[23] Biver T. (2013). Stabilisation of Non-Canonical Structures of Nucleic Acids by Metal Ions and Small Molecules. Coord. Chem. Rev..

[24] Sugimoto N., Wu P., Hara H., Kawamoto Y. (2001). PH and Cation Effects on the Properties of Parallel Pyrimidine Motif DNA Triplexes. Biochemistry.

[25] Litan A., Langhans S.A. (2015). Cancer as a Channelopathy: Ion Channels and Pumps in Tumor Development and Progression. Front. Cell. Neurosci..

[26] Nakano S., Miyoshi D., Sugimoto N. (2014). Effects of Molecular Crowding on the Structures, Interactions, and Functions of Nucleic Acids. Chem. Rev..

[27] Jain S., Kaur J., Prasad S., Roy I. (2021). Nucleic Acid Therapeutics: A Focus on the Development of Aptamers. Expert Opin. Drug Discov..

[28] Murakami K., Izuo N., Bitan G. (2022). Aptamers Targeting Amyloidogenic Proteins and Their Emerging Role in Neurodegenerative Diseases. J. Biol. Chem..

[29] Ozturk M., Nilsen-Hamilton M., Ilgu M. (2021). Aptamer Applications in Neuroscience. Pharmaceuticals.

[30] Amato J., Mashima T., Kamatari Y.O., Kuwata K., Novellino E., Randazzo A., Giancola C., Katahira M., Pagano B. (2020). Improved Anti-Prion Nucleic Acid Aptamers by Incorporation of Chemical Modifications. Nucleic Acid Ther..

